# The relationship between self-esteem and self-concept clarity is modulated by spontaneous activities of the dACC

**DOI:** 10.3389/fpsyg.2022.926181

**Published:** 2022-10-05

**Authors:** Yujie Chen, Yanchi Liu, Yuan Gao, Xin Wu, Lei Mo

**Affiliations:** ^1^School of Psychology, South China Normal University, Guangzhou, China; ^2^Centre for Studies of Psychological Applications, South China Normal University, Guangzhou, China; ^3^Guangdong Key Laboratory of Mental Health and Cognitive Science, South China Normal University, Guangzhou, China; ^4^Key Laboratory of Brain Cognition and Educational Science, Ministry of Education, South China Normal University, Guangzhou, China; ^5^School of Psychology, Xinxiang Medical University, Xinxiang, China

**Keywords:** ALFF, self-esteem, self-concept clarity, dorsal anterior cingulate cortex, resting-state fMRI

## Abstract

Although it has been found that self-esteem and self-concept clarity are positively correlated, self-determination theory shows that the positive relationship might be lowered for individuals whose basic psychological needs are chronically thwarted. The exact neural mechanisms underlying the relationship between self-esteem and self-concept clarity are still not fully understood. The dorsal anterior cingulate cortex (dACC) plays an important role in monitoring basic psychological needs, considering that it is more active when some basic psychological needs are actually or potentially thwarted. To better understand the neural mechanisms underlying the relationship between self-esteem and self-concept clarity, we investigated the differences in the relationship between self-esteem and self-concept clarity among healthy adults with different levels of spontaneous activities of the dACC using rs-fMRI combined with amplitude of low-frequency fluctuation (ALFF). As expected, the results showed that the positive relationship between self-esteem and self-concept clarity was modulated by the ALFF value of the right dACC, which indicated that the positive relationship was significant when the ALLF value of the right dACC was lower, but the positive relationship was not significant when the ALFF value of the right dACC was higher. The modulating roles of right dACC might also reflect that the individuals with higher ALFF value of dACC might experience chronically thwarted relatedness of basic psychological needs, which means the more disturbed by thwarting relatedness information in individuals, the lower positive relationship emerged.

## Introduction

Self-esteem is an attitude based on positive and negative self-evaluations (Rosenberg, 1965; Yang et al., 2016; Wu et al., 2017). Self-concept clarity refers to the degree to which the contents of self-concept are clearly and confidently defined, internally consistent, and temporarily stable (Campbell et al., 1996; Findley, 2013). Campbell (1990) found that individuals with high self-esteem have a more consistent or stable self-concept than those with low self-esteem, which means that self-concept clarity is positively related to self-esteem. However, the positive relationship is not always stable, since self-determination theory suggests that the stability and the internal consistency of the self-concept do not increase with increasing self-esteem when some basic psychological needs (autonomy, competence, and relatedness) are not met (Deci and Ryan, 1995; Ryan and Deci, 2017). The exact neural mechanisms underlying the relationship between self-esteem and self-concept clarity are still not fully understood.

The dorsal anterior cingulate cortex (dACC) plays an important role in monitoring basic psychological needs, considering that it is more active when some basic psychological needs are actually or potentially thwarted (Eisenberger et al., 2003, 2007; Slavich et al., 2010; Rotge et al., 2014); this also makes it a crucial area for modulating the relationship between self-esteem and self-concept clarity. Specifically, one of the functions of the dACC is related to conflict monitoring. The dACC deals with signaling the presence of conflicts in information processing and the need for higher cognitive control to resolve them, producing neural and behavioral adjustments (Kerns et al., 2004; Carter and van Veen, 2007). For example, the dACC is more active when some cues potentially indicate competence being negatively evaluated, such as negative feedback for task performance (Zanolie et al., 2008; van Schie et al., 2018) or acquiring a lower status relative to others (Yaple and Yu, 2020). Experiencing social exclusion that directly thwarts relatedness could also make the dACC more active (Eisenberger et al., 2003, 2007; Slavich et al., 2010; Rotge et al., 2014). Thus, the dACC plays an important role in monitoring the information that thwarts basic needs and then initiating cognitive control to resolve problems or regulate behaviors to improve performance. The difference in the activity level of the dACC might lead to a different relationship between self-esteem and self-concept clarity.

Resting-state fMRI (rs-fMRI) requires neither stimulation nor response and reflects the spontaneous neuronal activity or background neurophysiological processes of the human brain. Moreover, it has been found that the amplitude of low frequency (0.01–0.1 Hz) fluctuation (ALFF) of RS-fMRI can be used to investigate the functions of certain brain regions, which are consistent with the task-fMRI results to some extent (Biswal et al., 1995; Fox and Raichle, 2007). Accordingly, the spontaneous activities of the dACC, similar to functional activity, could reflect the monitoring of basic psychological needs, such as suffering the experience of social exclusion at some level.

One important type of thwarting relatedness is social exclusion, which has also been proven to be related to dACC (Rudolph et al., 2016). Consistent with this finding, previous studies have shown that the functions of brain networks, including the dACC, are increased when relatedness is chronically thwarted (e.g., suffering the experience of social exclusion) using resting-state functional magnetic resonance imaging (RS-fMRI) (van der Werff et al., 2013; Layden et al., 2017). Moreover, the spontaneous activities of certain brain regions may reflect their preparing roles in processing information when performing tasks (Fox and Raichle, 2007; Raichle and Snyder, 2007; Raichle, 2010). Therefore, we speculate that the spontaneous activities of the dACC might reflect relatedness being chronically thwarted (e.g., under social exclusion conditions), which means the dACC is sensitive to prepare to monitor thwarting relatedness information.

Previous evidence showed that the dACC was more active under situations of social exclusion for individuals whose relatedness is chronically thwarted (Will et al., 2015). In addition, the amplitude of low-frequency fluctuation (ALFF) of RS-fMRI was one index of spontaneous brain activities. The ALFF value of the dACC could reflect the level of monitoring basic psychological needs, such as suffering the experience of social exclusion. The value of the ALFF in the dACC was higher, and the feeling of thwarting relatedness was higher. According to self-determination theory, the positive relationship between self-esteem and self-concept clarity could be broken under the condition of basic psychological needs being thwarted (such as social exclusion). Therefore, regions of the dACC were selected based on the meta-analysis of social-pain fMRI studies (Rotge et al., 2014), and their ALFF values were then calculated to study the modulating roles of the dACC using the Rosenberg self-esteem scale (RSES) and self-concept clarity scale (SCCS) to measure self-esteem and self-concept clarity, respectively. In summary, the spontaneous activities of the dACC confirmed in monitoring social exclusion information might play modulating roles in the relationship between self-esteem and self-concept clarity.

To better understand the neural mechanisms underlying the relationship between self-esteem and self-concept clarity, we investigated the differences in the relationship of SCCS and RSES among healthy adults with different levels of spontaneous activities of the dACC using rs-fMRI combined with amplitude of low-frequency fluctuation (ALFF). Based on self-determination theory and the evidence from RS-fMRI studies, we hypothesized that the score of the SCCS might be positively correlated with the score of the RSES for individuals with lower ALFF values of the dACC, while the positive relationship might not be observed or lowered for individuals with higher ALFF values of the dACC.

## Methods

### Subjects

A priori power analysis (G*Power 3.1.9.2; Faul et al., 2009), setting the effect size |ρ| = 0.3 for the two-tailed test, indicated that a minimum of 109 individuals would provide the recommended 0.90 power level (Cohen, 1988). Thus, 123 undergraduate students (67 female, mean age = 18.31 ± 0.82) took part in the experiment. All participants were right-handed Chinese individuals. They were recruited through advertisements on bulletin boards at Xinxiang Medical University. The inclusion criteria were as follows: not diagnosed with a mental or neurological illness; no visual difficulties or history of alcohol or drug dependence; and no history of psychiatric disorders within three generations.

### Materials

The experiment used the Rosenberg self-esteem scale (RSES) to measure individual self-esteem (Rosenberg, 1965). There are 10 items on the SES, and participants were asked to rate the degree to which each item described them using a 4-point scale (“1” = strongly disagree, “2” = disagree, “3” = agree, and “4” = strongly agree). There are five positive scoring questions and five negative scoring questions in the questionnaire, with the total score ranging from 10 to 40 points. After reversing the score of the negative items, the RSES score was calculated by summing the scores of the 10 items, and the reliability of the RSES was 0.905, with a mean score of 27.92 ± 5.38 in our sample.

The SCCS was used to measure self-concept clarity (Campbell et al., 1996). There are 12 items in the SCCS, and each participant was asked to rate the degree to which each item described them using a 5-point scale (“1” = strongly disagree, “3” = uncertain, and “4” = strongly agree). Except for the 6th and 11th items, the others are reverse scored. After reversing the score of the reversing items, the score of the SCCS was calculated by averaging the scores of the 10 items, and the reliability of the SCCS was 0.832, with a mean of 3.78 ± 0.96 in our sample.

### RS-fMRI data acquisition

To acquire the RS-fMRI data, the subjects were asked to focus on the “+” in the center of the screen without moving their body or head. The RS-fMRI measure included 242 scans and was 484 s in duration.

Whole-brain RS-fMRI images were acquired from a Siemens 3T scanner (MAGENTOM Trio, a Tim system) with a gradient echo-planar imaging sequence: echo time (TE) = 30 ms; repetition time (TR) = 2,000 ms; flip angle = 90°; slices = 32; slice thickness = 3.0 mm; slice gap = 1 mm; field of view (FOV) = 220 × 220 mm^2^; resolution matrix = 64 × 64; in-plane resolution = 3.4 × 3.4 mm^2^; interslice skip = 0.99 mm. Thus, 242 functional images were acquired for each subject.

In addition, high-resolution T1-weighted anatomical images were acquired using a magnetization-prepared, rapid gradient echo sequence (repetition time = 1,900 ms; echo time = 2.52 ms; inversion time = 900 ms; flip angle = 9°; resolution matrix = 256 × 256; slices = 176; thickness = 1.0 mm; voxel size = 1 × 1 × 1 mm^3^).

### fMRI data analysis

#### Preprocessing

RS-fMRI images were preprocessed using SPM 12 (http://www.fil.ion.ucl.ac.uk/spm) and DAPABI 6.1 (DPABI: http://rfmri.org/dpabi) in MATLAB 8.1 (http://cn.mathworks.com/). First, the DICOM raw data were converted to NIFTI format, and at the same time, the first 10 images were discarded. Then, the remaining images were preprocessed by the following steps: slice-timed, realigned, and head-motion correction where the various covariates, such as white matter, cerebrospinal fluid, and the Friston 24-parameter were regressed out. Thereafter, all the functional images were normalized into the Montreal Neurological Institute (MNI) space of 3 × 3 × 3 mm^3^ voxel sizes. The normalized images were spatially smoothed with a Gaussian kernel having a full width at half maximum (FWHM) of 8 mm. Then, linear trends were removed, and the images were temporally bandpass filtered (0.01–0.08 Hz) to reduce low-frequency drift and high-frequency noise. Finally, the ALFF was calculated and transformed to *z*-values by using DPABI, which is an efficient method and is widely used in RS-fMRI studies (Zuo and Xing, 2014; Yan et al., 2016).

#### Region of interest analysis

According to a meta-analysis of task-fMRI study on social pain (Rotge et al., 2014), the coordinates of the bilateral dACC (−8, 18, 42; 8, 24, 24) were selected, and then the two ROIs were acquired by spheres with 6 mm radius peeking in each of these coordinates, whose ALFF values were extracted using the “ROI Signal Extractor” of DPABI. Finally, the modulating role of the mean ALFF value of each ROI in the relationship between the total RSES score and the mean SCCS score was analyzed using Model 1 of PROCESS 3.0 (Hayes, 2018), where sex and age were set as covariates.

#### Whole brain analysis

Whole-brain analysis of imaging data was performed using a two-sample *t*-test in SPM 12. To identify the brain regions where the interaction between RSES and SCCS existed, the sample was divided into the low-RSES group (lower than mean SES) and the high-RSES group (higher than mean SES) based on the mean score of RSES; then, the ALFF z-values of the low-RSES group and the high-HSES group were inserted into the two-sample *t*-test of SPM 12, and at the same time, the score of SCCS was defined as a covariate, where sex and age were also set as covariates. This method has been successfully employed in previous studies to analyze the interaction between variables and the index of structural and functional MRI (Takeuchi et al., 2013; Li et al., 2014, 2018; Wei et al., 2015). The interaction between RSES and SCCS was assessed using t-contrasts [(1, −1) or (-1 1)]. Statistical inference was *p* < 0.005 at the voxel level with Gaussian random field (GRF) corrected to *p* < 0.05 at the cluster level (Yan et al., 2016; Chen et al., 2018).

## Results

### Behavioral results

**Figure 1:**
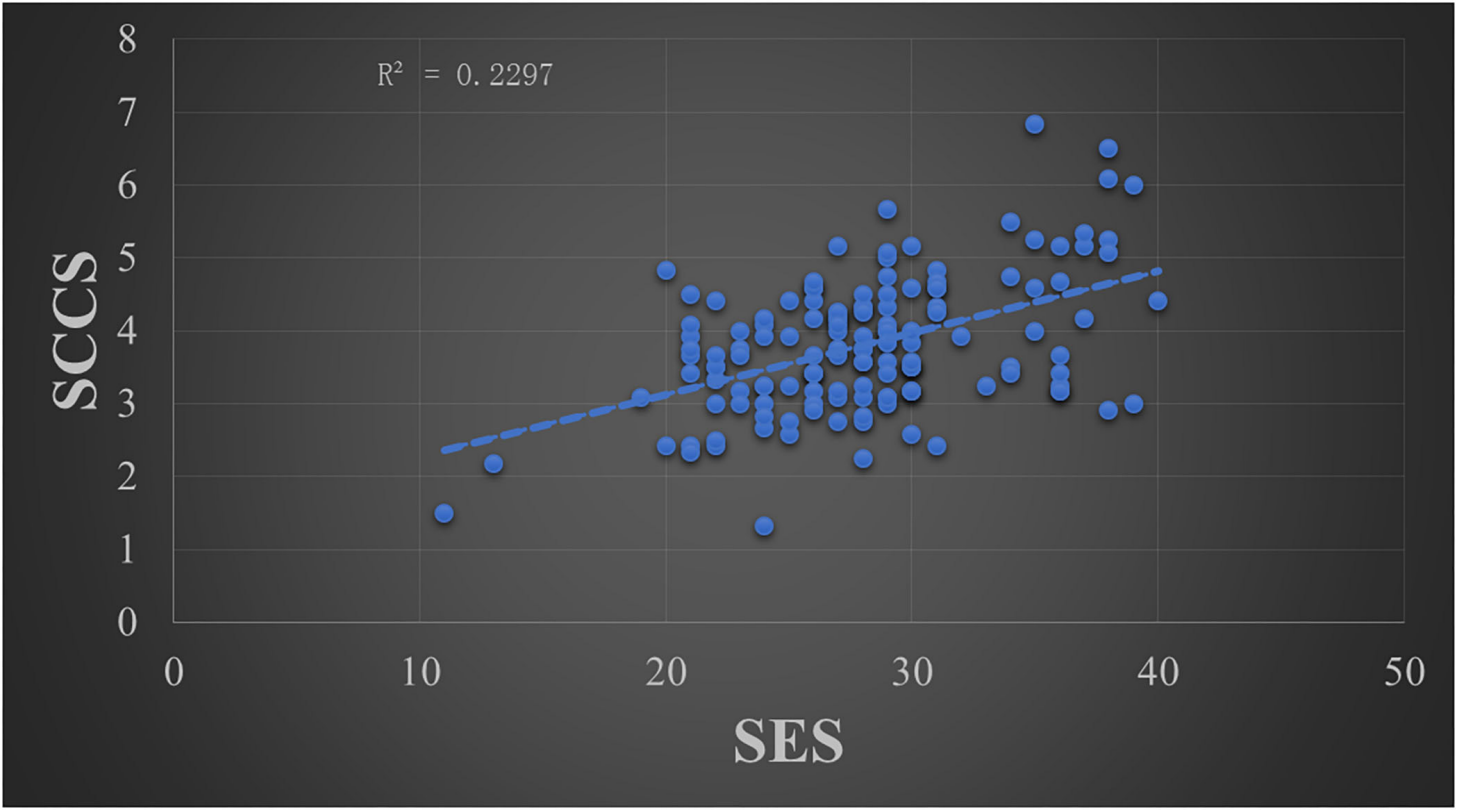
The relationship between the mean score of the SCCS and the total score of the Rosenberg self-esteem scale.

The skewness absolute values were 0.141 for the RSES score and 0.488 for the SCCS score, and the kurtosis absolute values were 0.371 for the RSES score and 0.713 for the SCCS score. According to previous studies (Curran et al., 1996; Xiao and Huang, 2022), the data met a normal distribution due to the skewness absolute values being less than 2 and the kurtosis absolute values being less than 7. Thus, the Pearson Product-Moment Correlation was used to analyze the relationship between the RSES and SCCS scores, where the statistical threshold was two-tailed *p* < 0.05. The results showed that there was a significant positive correlation between the SES scores and SCCS scores (*r* = 0.479, *p* < 0.001, *R*^2^ = 0.2297) (see Figure 1).

### Results of ROI analysis

**Figure 2:**
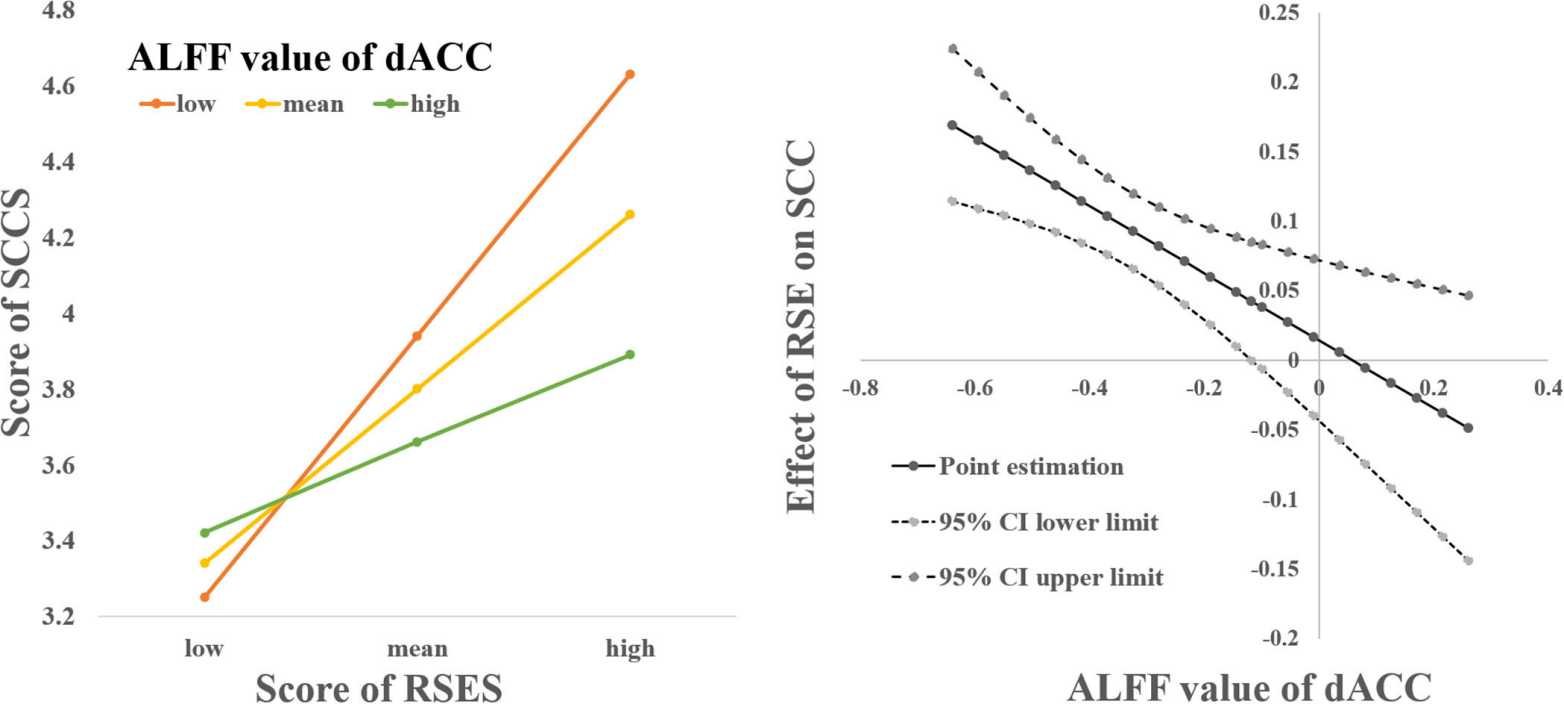
The relationship between SCCS and SES was modulated by spontaneous activation of the right dACC.

The interaction between the RSES score and the left dACC was not significant when predicting the SCCS score [*F*_(1, 117)_ = 2.876, *p* = 0.093, Δ*R*^2^ = 0.017]; however, the interaction between the RSES score and the right dACC score was significant when predicting the SCCS score [*F*_(1, 117)_ = 9.602, *p* = 0.002, Δ*R*^2^ = 0.055]. Simple slope analysis results show that when the ALFF value of the right dACC was lower (mean +1 SD), the RSES score was significantly positively correlated with the SCCS score (β = 0.694, t = 7.334, *p* < 0.001), while when the ALFF of the right dACC was higher (mean +1 SD), there was a significant correlation between the RSES and SCCS scores (β = 0.256, *t* = 2.072, *p* = 0.041). The Johnson-Neyman intervals showed that the RSES score was positively correlated with the SCCS score when the ALFF value of the right dACC was lower than −0.118 (including 86.99% of our sample), but the positive relationships between them were not significant when the ALFF value was greater than this value (see Figure 2).

### Results of whole-brain analysis

**Figure 3:**
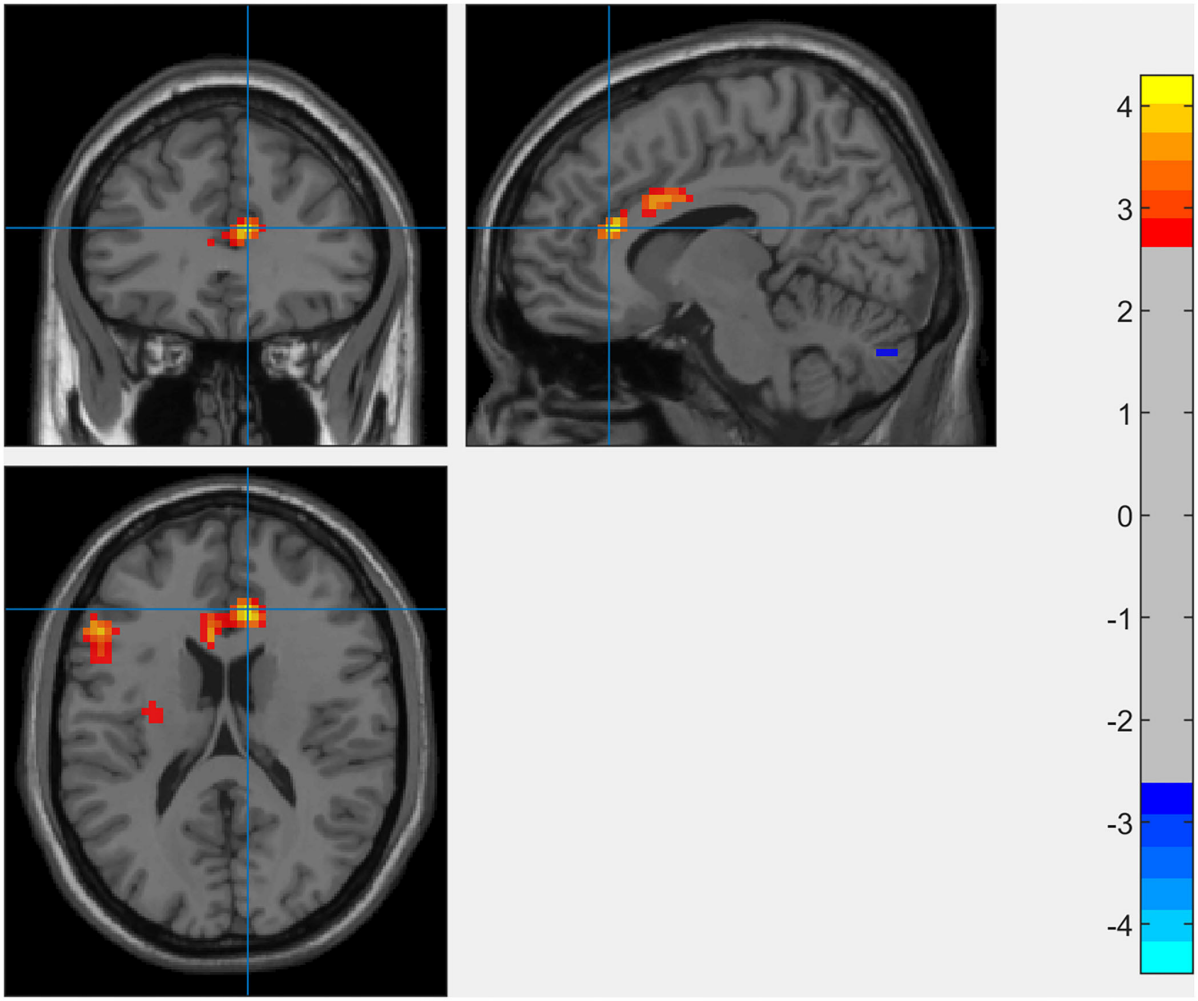
Whole-brain analysis of the interaction between SCCS and SES when predicting spontaneous activation of the brain (GRF-corrected *p* < 0.005 at the voxel level with *p* < 0.05 at the cluster level).

The results of the whole-brain analysis showed that the interaction effect between SCCS and RSES was significant in the right dACC (9, 33, 18) with [−1 1] t-contrast (see Figure 3 and Table 1).

**Table 1:** The relationships between SES and spontaneous activation of social brain regions identified by whole-brain analysis (GRF-corrected *p* < 0.005 in voxel level with *p* < 0.05 in cluster level).

**Cluster**	**Cluster size**	**Regions**	**BA**	***t*-value**	***p* (voxel level)**	**peek (x, y, z)**
1	**217**	**Right dACC**	**32**	**4.30**	**< 0.001**	**9, 33, 18**
		**Right dACC**	**24**	**4.06**	**< 0.001**	**6, 15, 27**
		Left corpus callosum		3.64	< 0.001	−6, 24, 15
2	314	Left IFG	45	4.04	< 0.001	−51, 24, 21
		Left Sub-Gyral		3.73	< 0.001	−39, −3, 24
		Left precentral	9	3.67	< 0.001	−48, 3, 30
3	228	Cerebellum posterior lobe		−3.81	< 0.001	27, −84, −33
		Cerebellum posterior lobe		−3.05	0.001	24, −60, −39
		Cerebellum posterior lobe		−2.89	0.002	42, −66, −36

IFG, inferior frontal gyrus.

## Discussion

The purpose of this study was to investigate the role of social exclusion dACC in modulating the relationship between self-esteem and self-concept clarity by using ALFF. The behavioral results showed that RSES scores were significantly positively correlated with the SCCS scores. RS-fMRI results showed that the ALFF value of the right dACC could modulate the relationship between RSES and SCCS. Specifically, there was a positive relationship between self-esteem and self-concept clarity when the ALFF value of the right dACC was lower, but the positive relationship was not significant when the ALFF value was higher.

The results of the present study showed that self-concept clarity was not associated with self-esteem when the ALFF value of the right dACC was higher. Self-determination theory suggests that relatedness is a basic psychological need (Deci and Ryan, 1995; Ryan and Deci, 2017). Furthermore, there is a gauge of the thwarted degree when the relatedness is thwarted (Leary and Baumeister, 2000; Leary et al., 2001), which might be related to dACC. Generally, it has been found that the dACC is a targeted region of relatedness being chronically thwarted, and it is therefore sensitive to the potentially thwarting-relatedness information (e.g., Eisenberger et al., 2003; van der Werff et al., 2013; Layden et al., 2017). The dACC is an important brain area, and different parts of it are responsible for different cognitive processes, such as conflict monitoring, contingent information processing, and social exclusion processing (Kerns et al., 2004). Although the present study is a resting state fMRI study and participants were not actually involved in a social exclusion experience, the ROI analysis is based on meta-analyses of task-fMRI studies in social exclusion, which makes it clear that the coordinates of the bilateral dACC (−8, 18, 42; 8, 24, 24) we used in the present study are mainly responsible for social exclusion (Rotge et al., 2014). In addition, it should be pointed out that although the peak coordinates (9, 33, 18 and 6, 15, 27) of the dACC found by using the whole brain analysis are different from the dACC found by the ROI (8, 24, 24), the dACC has 271 voxels which includes the ROI. Considering that the spontaneous activities of certain brain regions can reflect their preparing roles in processing information (Raichle and Mintun, 2006; Fox and Raichle, 2007; Raichle, 2010), the right dACC might be an inner gauge of relatedness, which makes it more active in the resting state and more sensitive to social exclusion when the ALFF value is higher. Thus, the results of the present study might support the self-determination theory, which implies that the positive relationship between self-concept clarity and self-esteem is lower when relatedness is chronically thwarted.

The modulating roles of the right dACC in this study might imply individual differences in the sensitivity of the dACC to the information that thwarts relatedness. Consistent with this, Eisenberger and Lieberman (2004) used the neural alarm system to interpret the roles of the dACC in social exclusion based on conflict monitoring theory (Kerns et al., 2004; Carter and van Veen, 2007), and suggested that there were individual differences in the sensitivity of the dACC to social exclusion, namely, the more responsive the dACC was, the more prone it was to monitor social exclusion information. Furthermore, previous research also showed that there are huge individual differences in the behavioral, physiological, and neural responses to social exclusion due to relatedness being chronically thwarted, such as lower peer popularity (Tobia et al., 2017), interpersonal competence (Masten et al., 2009), self-injury (Groschwitz et al., 2016), peer status (de Water et al., 2017), attachment (Liddell and Courtney, 2018), psoriasis (Ponsi et al., 2019), and emotional maltreatment (Schulz et al., 2022). Moreover, some of them also found that the dACC was more responsive to the exclusion information (Masten et al., 2009; de Water et al., 2017; Schulz et al., 2022). According to these results, the modulating roles of right dACC might also reflect that the positive relationship between self-concept clarity and self-esteem is not always stable and that there are individual differences, meaning that the positive relationship between self-concept clarity and self-esteem may be broken more easily in someone whose spontaneous activities of dACC are more active.

Although sociometer theory suggests that self-esteem is decreased when suffering social exclusion (Leary and Baumeister, 2000; Leary et al., 2001), the function of the right dACC was not directly associated with self-esteem based on our results, which is consistent with previous studies. For example, Blackhart et al. (2009) found that social exclusion did not affect self-esteem, although it did elicit some negative emotions. The results from neuroimaging studies also showed that the dACC was not related to self-esteem (Wu et al., 2017) or the distress elicited by social exclusion (Kross et al., 2007), but self-esteem was related to the functions of the dACC when processing self-related stimuli (Yang et al., 2012) and negative feedback on the self (Peng et al., 2019). However, it should be noted that self-esteem can be further divided into contingent and true self-esteem (Deci and Ryan, 1995; Ryan and Deci, 2017). Moreover, Deci and Ryan (1995) argue that although social exclusion can decrease the self-worth of individuals with contingent self-esteem, individuals with true self-esteem might not be influenced. Considering that the dACC is more sensitive to contingent information, we further thought that individuals with higher ALFF values in the right dACC could have contingent self-esteem, making them more easily influenced by contingent information. Because individuals with both true self-esteem and higher contingent self-esteem report higher scores on self-esteem scales (Deci and Ryan, 1995), the above inconsistent results might result from the true and contingent self-esteem not being distinguished by using the RSES.

In conclusion, this study explored whether the positive relationship between self-esteem and self-concept clarity was modulated by the spontaneous activation of the dACC using ALFF. The results showed that the ALFF value of the right dACC played a vital role in modulating the positive relationship. Although it is helpful for us to learn the neural mechanisms underlying the relationship between self-esteem and self-concept clarity, we acknowledge the following limitations. First, although there are many brain regions related to basic psychological needs (Cacioppo et al., 2013; Rotge et al., 2014; Vijayakumar et al., 2017), the present study only investigated the modulating roles of the dACC. Thus, the roles of the other brain regions in the relationship should be studied in the future. Second, we thought that the right dACC might be a critical region to divide true and contingent self-esteem based on our results, yet direct evidence is still absent. In the future, the role of the right dACC in the processing of contingent information should be considered (van Schie et al., 2018; Yaple and Yu, 2020; van Houtum et al., 2021; Tan et al., 2022), which might help uncover the neural basis of the relationships between self-esteem and self-concept clarity (e.g., Deci, 1971; Deci et al., 1999).

## Data availability statement

The original contributions presented in the study are included in the article, further inquiries can be directed to the corresponding authors.

## Ethics statement

The studies involving human participants were reviewed and approved by the Ethics Committee of the Xinxiang Medical University. The patients/participants provided their written informed consent to participate in this study.

## Author contributions

YC and XW developed the conception of the study, analyzed the data, and drafted the manuscript. YL and YG participated in some revision work. LM provided critical revisions in the final version of the manuscript. XW and LM gave important advice throughout the whole study. All authors contributed to manuscript revision, read, and approved the submitted version.

## Funding

This work was supported by grants from the National Social Science Foundation of China (19ZDA360), the Guangdong Basic and Applied Basic Research Foundation (2021A1515011259), the fellowship of China Postdoctoral Science Foundation (2020M672660), and the Science and Technology Projects in Guangzhou (202201010626).

## Conflict of interest

The authors declare that the research was conducted in the absence of any commercial or financial relationships that could be construed as a potential conflict of interest.

## Publisher's note

All claims expressed in this article are solely those of the authors and do not necessarily represent those of their affiliated organizations, or those of the publisher, the editors and the reviewers. Any product that may be evaluated in this article, or claim that may be made by its manufacturer, is not guaranteed or endorsed by the publisher.
